# Supplementary material for: FABP4-mediated lipid accumulation and lipolysis in tumor-associated macrophages promote breast cancer metastasis
Source: eLife. 2024 Nov 8;13:RP101221. doi: 10.7554/eLife.101221 (PMC11548877; doi:10.7554/eLife.101221)
Supplement: Supplementary file 2. [file elife-101221-supp2.docx]

| **Supplement File 2. Summary statistics for categorical variables and their association with outcome variables** | | | | | | | |
| --- | --- | --- | --- | --- | --- | --- | --- |
| Variable | Level | N = 59 (%) | CD163+ | | FABP4+ CD163+ | |  |
|  |  |  | Median (IQR) | p-value | Median (IQR) | p-value |  |
| Age | ≤65 | 22 (37.3%) | 835.5 (501.75, 1499.25) | 0.589 | 57 (10.25, 132.5) | 0.583 |  |
|  | >65 | 37 (62.7%) | 887 (350, 1605) |  | 29 (10, 105) |  |  |
| BMI | <30 | 28 (47.5%) | 866 (304, 1211.75) | 0.192 | 39 (5.75, 114.5) | 0.832 |  |
|  | ≥30 | 31 (52.5%) | 845 (418.5, 1908) |  | 55 (10.5, 98.5) |  |  |
| Vital status | 0 | 50 (84.7%) | 835.5 (350, 1484.5) | 0.096 | **33.5 (10, 74.75)** | **0.043** |  |
|  | 1 | 9 (15.3%) | 1449 (586, 2726) |  | **159 (84, 189)** |  |  |
| Elston Ellis grade | 1 | 14 (23.7%) | 540.5 (232.75, 1108.75) | 0.098 | 10 (0.75, 49) | **0.027** |  |
|  | 2 | 29 (49.2%) | 736 (468, 1721) |  | 47 (11, 105) |  |  |
|  | 3 | 16 (27.1%) | 1191.5 (753.5, 1780.5) |  | 72.5 (26.75, 154) |  |  |
| Focality | 1 | 54 (91.5%) | 946.5 (354.75, 1671.75) | 0.663 | 46 (10.25, 116) | 0.114 |  |
|  | >1 | 5 (8.5%) | 826 (529, 845) |  | 10 (0, 47) |  |  |
| Metastasis | 0 | 46 (78%) | 618.5 (314.5, 1181.5) | **<0.001** | 20 (4.25, 67.75) | **<0.001** |  |
|  | >0 | 13 (22%) | 1929 (1516, 2726) |  | 187 (71, 238) |  |  |
| Lymphovascular invasion | 0 | 52 (88.1%) | 781 (350, 1484.25) | 0.206 | 39 (9, 84.75) | 0.113 |  |
|  | 1 | 7 (11.9%) | 1516 (977, 1663) |  | 117 (59, 165) |  |  |
| Hormone status | ER+ HER2- | 41 (69.5%) | 736 (350, 1363) | 0.206 | 38 (5, 87) | **0.026 *** |  |
|  | ER+ HER2+  ER- HER2+ | 3 (5.1%)  5 (8.5%) | 350 (329, 1035.5)  1274 (826, 2726) |  | 14 (7, 15.5)  69 (10, 76) |  |  |
|  | Triple Negative | 10 (16.9%) | 1527 (836.5, 2287.25) |  | 138 (60.25, 194.5) |  |  |

*, ER+ HER2- vs. Triple Negative, p = 0.007567; ER+ HER2+ vs Triple Negative, p = 0.006993; ER- HER2+ vs Triple Negative, p = 0.2544;
