# Supplementary material for: FABP4-mediated lipid accumulation and lipolysis in tumor-associated macrophages promote breast cancer metastasis
Source: eLife. 2024 Nov 8;13:RP101221. doi: 10.7554/eLife.101221 (PMC11548877; doi:10.7554/eLife.101221)
Supplement: Supplementary file 3. [file elife-101221-supp3.docx]

| Glycerol-3-Phosphate Acyltransferase 1 (Gpam1) | GTGGCTTCCTCGAACGATTA | CTGCCATCCATCCCAGATATAC |
| --- | --- | --- |
| Glycerol-3-Phosphate Acyltransferase 3 (Gpam3) | GATGAGTCACCCATGGAGAAAG | CAGAGGTAGCAGGAAGCAATAG |
| Glycerol-3-Phosphate Acyltransferase 4 (Gpam4) | TCACCAGAGGAGATAGGAAGAG | CCCAGACCTGAAGATGGAATATC |
| 1-acylglycerol-3-phosphate O-acyltransferase 2 (Agpat2) | CAAAGTGTGGATCTACCCAGAG | GGCTCCTTAATGGCAGAGTT |
| Lipin 1a | GCTCAAGGCTGGGCTATTTA | GAGGCTCTTTGGACTGTCTATC |
| Diacylglycerol O-acyltransferase 1 (Dgat1) | CAGCTCAGACAGTGGTTTCA | GGCTTCATGGAGTTCTGGATAG |
| Diacylglycerol O-acyltransferase 2 (Dgat2) | GCTGACCACCAGGAACTATATC | GCACTCAAGAACTCGGTAGAA |
| CCAAT/enhancer-binding protein alpha (C/ebpα) | GCCCGGCAGTACTAGTATTAAG | TGGGACACAGAGACCAGATA |
| CCAAT/enhancer-binding protein beta (C/ebpβ) | CCTTTAGACCCATGGAAGTGG | CATCTTGTACTCGTCGCTCAG |
| Fatty Acid Binding Protein 4 (Fabp4) | TTTCCTTCAAACTGGGCGTG | CATTCCACCACCAGCTTGTC |
| Fatty Acid Binding Protein 5 (Fabp5) | AACCGAGAGCACAGTGAAG | ACACTCCACGATCATCTTCC |
| Beta 2 Adrenergic receptor 2 (Adrb2) | GAGCCTGCTGACCAAGAATAA | GAAGACCCGGGAATAGACAAAG |
| Beta 4 Adrenergic receptor 4 (Adrb4) | CTACAGCATCACCTTCCTTCTC | GGTTCTCCATTGTCTCAGTCTC |
| Patatin Like Phospholipase Domain Containing 2 | CCACTTTAGCTCCAAGGATGAG | TTCGAGAGGCGGTAGAGATT |
| Hormone Sensitive Type, Lipase E (Lipe) | AGGAGTCCCTATCTTCTCCATC | TCTGCCTCTGTCCCTGAATA |
| Acyl-CoA Synthetase Long Chain 1 (Acs1) | TGCCAGAGCTGATTGACATTC | GGCATACCAGAAGGTGGTGAG |
| Carnitine Palmitoyltransferase 1a (Cpt1a) | GTCGGTGAGCCTGGCCT | CTTGAGTGGTGACCGAGTCT |
| Carnitine Palmitoyltransferase 1b (Cpt1b) | TGCCTCTATGTGGTGTCCAA | CATGGCTTGTCTCAAGTGCT |
| 3-Hydroxy-3-Methylglutaryl-CoA Reductase (HMG-CoA) | CCAGAAGCTTTCGTCAGTAGAG | GCTCCCATCACCAAGGAATAA |
| Acetyl-CoA Acetyltransferase 1 (Acat1) | GTCCACCAAGCCAACATAGA | ACTGACGGACAAAGCTACAC |
| Acetyl-CoA Acetyltransferase 2 (Acat2) | CTGGAGGCATGGAGAATATGAG | CAGTTCCTGTCCCATCAGTAAG |
| CD36 Molecule (Cd36) | CAAAACGACTGCAGGTCAAC | CAATCCCAAGTAAGGCCATC |

**Supplement File 3. Realtime PCR primer sequences**
